# The Immediate and Sustained Positive Effects of Meditation on Resilience Are Mediated by Changes in the Resting Brain

**DOI:** 10.3389/fnhum.2019.00101

**Published:** 2019-03-26

**Authors:** Seoyeon Kwak, Tae Young Lee, Wi Hoon Jung, Ji-Won Hur, Dahye Bae, Wu Jeong Hwang, Kang Ik K. Cho, Kyung-Ok Lim, So-Yeon Kim, Hye Yoon Park, Jun Soo Kwon

**Affiliations:** ^1^Department of Brain and Cognitive Sciences, Seoul National University College of Natural Sciences, Seoul, South Korea; ^2^Institute of Human Behavioral Medicine, Seoul National University College of Medicine (SNU-MRC), Seoul, South Korea; ^3^Department of Psychology, Daegu University, Gyeongsan, South Korea; ^4^Department of Psychology, Chung-Ang University, Seoul, South Korea; ^5^Department of Psychiatry, National Institute of Forensic Psychiatry, Ministry of Justice, Gongjusi, South Korea; ^6^Department of Psychology, Duksung Women’s University, Seoul, South Korea; ^7^Department of Psychiatry, Seoul National University College of Medicine, Seoul, South Korea

**Keywords:** resilience, meditation, mindfulness training, templestay, resting-state functional connectivity

## Abstract

While recent studies have explored the maintenance of the effect of meditation on stress resilience, the underlying neural mechanisms have not yet been investigated. The present study conducted a highly controlled residential study of a 4-day meditation intervention to investigate the brain functional changes and long-term effects of meditation on mindfulness and resilience. Thirty participants in meditation practice and 17 participants in a relaxation retreat (control group) underwent magnetic resonance imaging scans at baseline and post-intervention and completed the Cognitive and Affective Mindfulness Scale (CAMS) and Resilience Quotient Test (RQT) at baseline, post-intervention, and the 3-month follow-up. All participants showed increased CAMS and RQT scores post-intervention, but only the meditation group sustained the enhancement after 3 months. Resting-state functional connectivity (rsFC) between the left rostral anterior cingulate cortex (rACC) and the dorsomedial prefrontal cortex (dmPFC), precuneus, and angular gyrus was significantly increased post-intervention in the meditation group compared with the relaxation group. The changes in rACC-dmPFC rsFC mediated the relationship between the changes in the CAMS and RQT scores and correlated with the changes in the RQT score both immediately and at 3 months post-intervention. Our findings suggest that increased rACC-dmPFC rsFC *via* meditation causes an immediate enhancement in resilience that is sustained. Since resilience is known to be associated with the preventative effect of various psychiatric disorders, the improvement in stress-related neural mechanisms may be beneficial to individuals at high clinical risk.

## Introduction

Resilience is defined as an individual’s capacity for recovery after significant adversity. Recent studies have suggested the importance of strengthening individuals’ resilience through appropriate interventions (Padesky and Mooney, 2012; van der Werff et al., 2013; Vanhove et al., 2015). For individuals, especially those at risk of mental disorder, enhanced resilience helps protect against their clinical conversion due to various stressful events (Ludwig and Kabat-Zinn, 2008; Creswell and Lindsay, 2014; Goyal et al., 2014). Moreover, resilience serves as a framework to promote stable remission in individuals with psychiatric disorders and to attenuate symptom severity in patients with inadequate responses to medication (Segal et al., 2010; Hoge et al., 2013; Waugh and Koster, 2015; Sharma et al., 2016). In this context, meditation has been shown to exert psychological and physiological effects on stress resilience and self-awareness and has been acknowledged as a promising adjunctive treatment in a broad range of psychiatric patients (Rubia, 2009; van der Velden and Roepstorff, 2015). However, most previous studies, which adopted meditation intervention for a few weeks, included possible limitations, such as isolation of the effects of meditation from other confounding factors or difficulty for subjects to regularly participate in the intervention. Therefore, recent studies have adopted several-day intensive residential meditation retreats designed to maximize training compliance and minimize the effects of non-meditative factors (Tang et al., 2007, 2015; Xue et al., 2011; Hwang et al., 2017).

Despite the growing interest in meditation and the significant progress achieved in understanding the associated dynamic neural changes *via* meditation (Lazar et al., 2005; Tang et al., 2015, 2007; Jang et al., 2011; Kang et al., 2012), only a few studies have focused on the maintenance of the effect. According to studies involving several months of follow-up after the intervention, both the meditation and active control groups exhibit improvements in stress-related psychological or physiological characteristics immediately after the intervention, but the effect is maintained only in the meditation group (Epel et al., 2016; Hwang et al., 2017). These findings suggest that meditation practices may induce changes in neural mechanisms to maintain the improvement, but the putative neural correlates underlying the maintenance of the effect have not yet been explored in neuroimaging studies.

According to recent meta-analyses, changes in anterior cingulate cortex (ACC) activation is consistently reported under various types of meditation and in short-term meditation studies (Bush et al., 2000; Fox et al., 2016). The ACC functions as a nexus of information processing, and 1 month of 30-min meditation interventions were shown to significantly increase the network efficiency and connectivity of the ACC in terms of resting-state functional connectivity (rsFC; Bush et al., 2000; Xue et al., 2011). However, the ACC can be differentiated into the rostral ACC (rACC) and the caudal ACC (cACC) based on its cytoarchitecture and functional connectivity. In general, the rACC appears to mainly be involved in the default mode network (DMN) and affective system, while the cACC is related to the sensorimotor and attention network (Milham and Banich, 2005; Margulies et al., 2007; Mohanty et al., 2007; Habas, 2009). Despite its distinguishing features, the majority of studies on the effects of meditation on the brain have not addressed its subregions. Moreover, only a few studies have explored the neural mechanism underlying the improvement of resilience through meditation. Although a recent study identified a decrease in subgenual ACC-amygdala rsFC, the region was associated with the perceived stress level based on region of interest (ROI)-wise rsFC analysis, and such an ROI-based approach limits the understanding of changes in resilience in the whole brain (Taren et al., 2015). Therefore, the neural correlates of resilience that are altered by short-term meditation remain unknown.

Motivated by previous findings, we sought to investigate whether changes in the rsFC contribute to improved resilience and its maintenance after 4 days of intensive residential meditation. Hence, we estimated whole-brain seed-to-voxel rsFC with ACC subregions and explored group-by-time interactions in rsFC maps for each ACC subregion. We hypothesized that: (1) the meditation group would present significant changes in rACC functional connectivity after the intervention; and that (2) these neural changes would be associated with improved resilience both immediately and at the 3-month follow-up.

## Materials and Methods

### 4-Day Intensive Meditation (Templestay) Project

**Figure 1:**
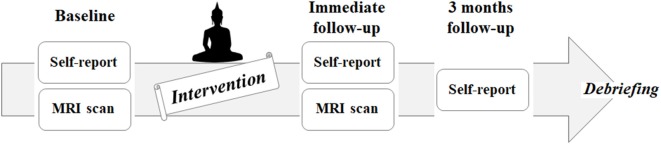
Flowchart of the intervention study, including enrollment, allocation, baseline, follow-up, and analysis.

This project is aimed to investigate the behavioral and neural effects of three nights and 4 days of intensive meditation (Templestay program) using multidimensional approaches, including self-reported questionnaires, behavioral tasks, and multiple brain imaging modalities. The data used in the current study are part of the Templestay project. This study was carried out in accordance with the recommendations of Institutional Review Board of Seoul National University Hospital Committee with written informed consent from all subjects. All subjects gave written informed consent in accordance with the Declaration of Helsinki. The protocol was approved by the Institutional Review Board of Seoul National University Hospital Committee. To minimize the placebo effect, all participants were informed about two types of Templestay programs—a Buddhism meditation type and a relaxation type—prior to participation. Those who were willing to be assigned to either type were enrolled and randomly allocated to either an intensive meditation group (MED) or the control group using a computerized algorithm based on 2:1 mixed block randomization (44 participants in the MED group, 23 participants in the control group). The brain imaging data were collected at two time points, baseline and immediately after the intervention, while self-report questionnaires were measured at three time points: baseline, immediate follow-up, and at a 3-month follow-up (Figure 1). The recruitment strategy, intervention program, and results from the self-report questionnaires were described thoroughly in a previous report (Hwang et al., 2017). A brief introduction to the materials is included in the Supplementary data and Supplementary Figure S1.

### Image Data Acquisition

Image data were acquired from all participants 2 to 3 days before and after the intervention using a 3T Trio scanner (Siemens Medical Systems, Erlangen, Germany) with a 32-channel head coil. All participants were asked to relax with their eyes open during the resting functional magnetic resonance imaging (fMRI) scan [repetition time (TR) = 2000 ms, echo time (TE) = 30 ms, 64 × 64 matrix, total of 150 volumes, one run, field of view (FOV) = 220 mm, flip angle (FA) = 80°, voxel size = 3.4 × 3.4 × 3.4 mm^3^, 34 slices, and no gap]. To ensure that the participants stayed awake, they were reminded to stay awake immediately before resting fMRI acquisition and were observed during acquisition through an eye-tracker in the MRI scanner. Additionally, high-resolution T1-weighted anatomical images were acquired with three-dimensional magnetization-prepared rapid gradient echo [TR = 1,670 ms, TE = 1.89 ms, FOV = 250 mm, flip angle (FA) = 9°, number of slices = 208, voxel size = 1.0 × 1.0 × 1.0 mm^3^].

### Analyses of Psychological Measures

To assess individuals’ resilience and mindfulness, all participants completed the modified Korean version of the Resilience Quotient Test (RQT) and the Cognitive and Affective Mindfulness Scale (CAMS)-Revised at three time points: baseline (T0), immediately after (T1), and 3 months after the intervention (T2). The RQT, developed by Reivich and Shatté (2002), is an inventory consisting of 27 items rated on a 5-point Likert scale, ranging from 1 (not at all) to 5 (very much so). CAMS measures everyday mindfulness, focusing mainly on the degree of participants’ thoughts and feelings on their experiences (Feldman et al., 2007). It consists of 12 items and assesses the core characteristics of mindfulness: the ability to regulate attention and orientation, to be aware, and to be nonjudgmental towards experience. Each item is rated on a 4-point scale, ranging from 1 (rarely/not at all) to 4 (almost always).

Independent samples *t*-tests were conducted for each scale to compare the differences between the groups at each time point. The group-by-time interaction of each scale was analyzed with a 3 [time (baseline/immediate follow-up/3-month follow-up), within-subject factor]-by-2 (group, between-subject factor) mixed analysis of variance (ANOVA) in SPSS, version 23.0 (IBM). To examine the directions of changes, *post hoc* *t*-tests were also conducted. The short-term effects on psychological measures were estimated with the scores from T0 and T1, while the long-term effects were estimated with the scores from T0 and T2.

### Functional Image Analysis

After discarding the first two scans, FMRIB Software Library (FSL^1^) was used to preprocess the image data. fMRI data analyses were performed with the following preprocessing steps: removal of nonbrain structures, slice-timing and motion correction, registration to T1-weighted images and spatial normalization to 2 mm the Montreal Neurological Institute (MNI) template using FMRIB’s Linear Image Registration Tool (FLIRT). In terms of mean head motion parameters, neither significant group differences nor time differences were observed (group comparison: *t* = −0.270, *p* = 0.788; time comparison in the MED group: *t* = −0.049, *p* = 0.961; time comparison in the control group: *t* = −1.704, *p* = 0.101; Van Dijk et al., 2012; Zeng et al., 2014b). Using a general linear model (GLM), we removed the effects of nuisance covariates, including six head motion parameters, and signals from cerebrospinal fluid and white matter segmented T1-weighted images. Residual images were spatially smoothed using a Gaussian kernel of full width at half maximum (4 mm) and were temporally smoothed using a high-pass filter of a Gaussian-weighted least-squares line fitted with sigma = 100.

According to previous neuroimaging studies on the effects of aging, we only included brain data from young adults (age 40 or younger) in the current study (Ferreira and Busatto, 2013); in this study, 34 participants were included in the MED group and 19 in the control group. Among the remaining 52 participants, data from five participants were excluded due to excessive head motion (>3 mm for x, y, z planes, *n* = 4) and image distortion during acquisition (*n* = 1). As a result, we included data from 30 participants in the MED group and 17 participants in the control group in the final analysis.

We then defined four seed regions, the rostral and cACC in each hemisphere, based on the Destrieux atlas (Destrieux et al., 2010). Next, we calculated the mean time series of each seed region and produced individual subject-level maps of all positively correlated voxels for each seed. Due to the debate about negative rsFC, we specifically focused on the positive connectivity maps (Fox et al., 2009; Murphy et al., 2009). To examine the group-by-time effect of the intervention, second-level analyses were carried out using GLM analysis. A one-sample *t*-test and a paired *t*-test for each group were also conducted to clarify our assumption regarding the distinctive rsFC of rACC and cACC and to investigate the effect of each intervention. Multiple comparisons for all the above mentioned statistical analyses were carried out at the cluster level using Gaussian random field theory (*Z* > 2.3; cluster significance; *p* < 0.010, corrected).

### Correlation and Mediation Analyses

To evaluate the hypothesis regarding the relationship between changes in functional connectivity and mindfulness and resilience, Pearson’s *r* correlation analysis was performed using SPSS v23.0. In the MED group, one participant at baseline and three participants at the 3-month follow-up were not available and were therefore excluded from the subsequent analyses. Normality and outliers in the raw data were checked prior to the analyses. In the correlation analysis, the strength of individual connectivity based on the significant clusters identified *via* the above rsFC analysis was extracted, and its variation was compared with the results of the self-reported questionnaires, RQT and CAMS. Both short (T0 to T1) and long-term (T0 to T2) improvements were analyzed based on the rsFC results. A *p*-value < 0.05 was considered statistically significant.

The mediation analysis test evaluated whether the association between *X* (predictor) and *Y* (outcome variable) was significantly reduced by including *M* (mediator) in the model when compared with the total relationship between *X* and *Y*. Thus, we further examined whether the association between the changes in mindfulness (*X*) and those in resilience (*Y*) could be explained *via* the indirect pathway based on the neural changes observed according to rsFC (*M*) using Mediation Toolbox^2^. Three variable-path models with bootstrap tests (10,000 iterations) were assessed.

### Analyses of the Effect of Religion

Since the meditation used in the study is based on Buddhism, the religious preferences of participants were collected to examine the potential effect of religion on the study result. Participants reported their religion among the following choices: Buddhist, Catholic, Presbyterian, none, or other.

## Results

### Demographic Characteristics

The demographic data for each group are summarized in Table 1. There were no significant differences detected in terms of demographic characteristics, including the age, sex, handedness, years of education, marital status, religion, or socioeconomic status of the participants or their parents.

**Table 1:** Demographic and clinical characteristics.

Variables	Meditation (*n* = 30)	Control (*n* = 17)	Statistical differences	
			*χ*^2^, *F* or *T*	*p*-value
Sex (male/female)	6/24	5/12	1.34	0.25
Handedness (right/left)^†^	26/4	17/0	2.48	0.12
Age (years, ± SD)	30.63 ± 4.97	31.71 ± 5.02	0.62	0.54
Education (years, ± SD)	16.53 ± 1.51	17.26 ± 1.62	−1.62	0.11
Religion (%)			0.34	0.57
None	21 (70.0%)	12 (70.6%)		
Buddhism	5 (16.7%)	2 (11.8%)		
Catholic	2 (6.7%)	2 (11.8%)		
Presbyterian	2 (6.7%)	1 (5.9%)		
Socioeconomic status (mean, ± SD)^‡^				
Participants’	2.60 ± 0.56	2.53 ± 0.64	0.52	0.47
Participants’ parents	2.57 ± 0.82	2.74 ± 0.96	0.79	0.38
RQT (mean, ± SD)^§^				
Baseline	90.83 ± 17.28	96.59 ± 0.30	3.19	0.08
Immediate follow-up	103.93 ± 13.27	103.76 ± 12.56	0.01	0.93
3-month follow-up	101.41 ± 17.65	98.53 ± 10.50	5.13	<0.05
CAMS (mean, ± SD)^§§^				
Baseline	28.40 ± 6.67	30.29 ± 4.43	3.35	0.07
Immediate follow-up	33.00 ± 6.59	32.88 ± 4.61	2.39	0.13
3-month follow-up	32.19 ± 6.26	30.88 ± 4.64	3.30	0.08

*^†^Annett Handedness scores. ^‡^Hollingshead socioeconomic status index, where 1–2 = high, and 4–5 = low. ^§^Korean version of the Resilience Quotient Test. ^§§^Cognitive and Affective Mindfulness Scale-Revised*.

### Psychological Measures

The RQT and CAMS scores of both groups at three time points are provided in Table 1. The baseline RQT and CAMS scores of the MED group were lower than those of the control group, but not significantly lower (*t* = 3.19, *p* = 0.08, and *t* = 3.35, *p* = 0.07, respectively).

To test the short and long-term effects of the intervention on each psychological measure more objectively, group-by-time mixed ANOVAs were conducted. A significant group-by-time interaction (*F*_(1,47)_ = 5.021, *p* = 0.031, and *F*_(1,47)_ = 5.913, *p* = 0.020, respectively) and an effect of time (*F*_(1,47)_ = 8.070, *p* = 0.007, and *F*_(1,47)_ = 2.903, *p* = 0.096, respectively) were observed, but no effect of the group (*F*_(1,47)_ = 0.615, *p* = 0.328, and *F*_(1,47)_ = 0.129, *p* = 0.722, respectively) was observed. In the *post hoc* *t*-test, both groups demonstrated significant increases of RQT and CAMS scores in the T0 to T1 (MED group: *t* = 4.734, *p* < 0.001, and *t* = 3.532, *p* < 0.005, respectively/control group: *t* = −3.676, *p* < 0.005, and *t* = −2.826, *p* < 0.005, respectively). In the T0 to T2 comparison, only the MED group presented significant increases in both questionnaires (MED group: *t* = −3.567, *p* < 0.005, and *t* = −2.910, *p* < 0.001, respectively/control group: *t* = −0.955, *p* = 0.354, and *t* = −1.000, *p* = 0.332, respectively). In the T1 to T2 comparison, only the control group showed significant decreases in both questionnaires (MED group: *t* = 1.329, *p =* 0.189, and *t* = 0.203, *p* = 0.841, respectively/control group: *t* = 2.217, *p* < 0.05, and *t* = 2.309, *p* < 0.05, respectively).

### Functional Connectivity Maps to Each Seed

**Figure 2:**
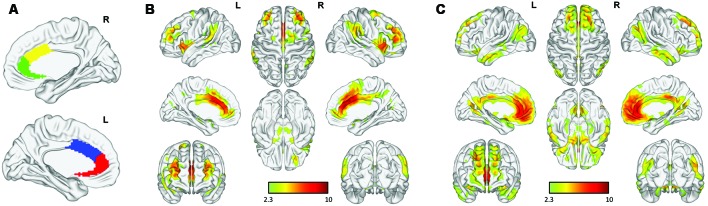
Functional connectivity of anterior cingulate cortex (ACC) subregions: **(A)** rostral (green, red) and caudal (yellow, blue) ACC in each hemisphere. Distinct functional connectivity was observed in the bilateral **(B)** caudal ACC (cACC) and **(C)** rostral ACC (rACC).

As shown in Figure 2, the cACC and rACC seed regions exhibited distinct rsFC patterns based on network cortical maps (Yeo et al., 2011). Bilateral rACC seeds were largely connected with regions (medial prefrontal cortex, precuneus, angular gyrus, amygdala, and hippocampus) belonging to the limbic network and DMN, whereas bilateral cACC seeds were linked with regions (dorsolateral prefrontal cortex, inferior frontal gyrus, inferior parietal lobule, and supplementary motor area) belonging to the fronto-parietal network and the sensorimotor network.

### Results of Group Analysis of Functional Connectivity Maps

**Figure 3:**
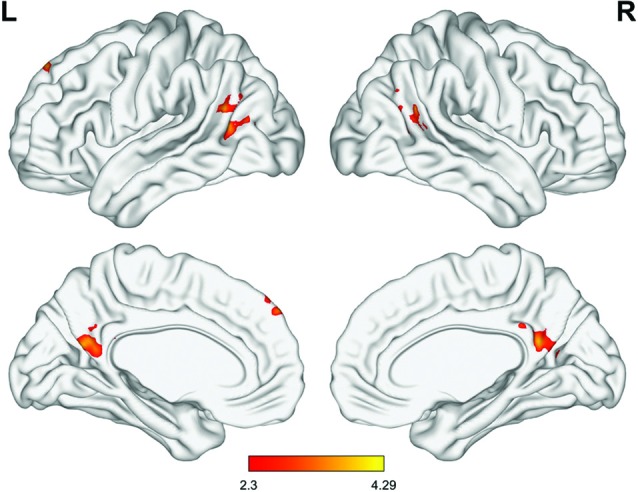
Significant differences in the group-by-time interaction effect (meditation group > control group, minimum *Z* score >2.3, cluster significance: *p* < 0.05, corrected). Significant differences were observed in connectivity involved in the default mode network (DMN), including the left rACC, dorsomedial prefrontal cortex (dmPFC), precuneus and temporoparietal junction (TPJ).

Among the four seed regions, only the left rACC showed significant group-by-time interactions, in several regions. Compared with the control group, the MED group presented increased rsFC between the left rACC and the dorsomedial prefrontal cortex (dmPFC), precuneus and angular gyrus in the MED group at the immediate post-intervention follow-up, whereas there was no significant group difference in rsFC for this seed region at baseline (Figure 3).

To further investigate the effect of each intervention on the brain functional network for an exploratory purpose, paired *t*-tests were performed for each group. In the MED group, the rsFC between the left rACC and the regions involved in the DMN, including the dmPFC, precuneus, and angular gyrus, was significantly increased after the intervention. The rsFC of the right cACC with the posterior cingulate cortex (PCC) was also significantly increased after the intervention. No additional significant changes in rsFC with the right rACC and left cACC were observed. In the control group, bilateral rACC rsFC with the temporal gyrus was increased, while the rsFC of the right rACC with the thalamus was decreased after the intervention. No significant changes were observed in rsFC with the bilateral cACC (Supplementary Figure S2).

### Correlation and Mediation Analyses

**Figure 4:**
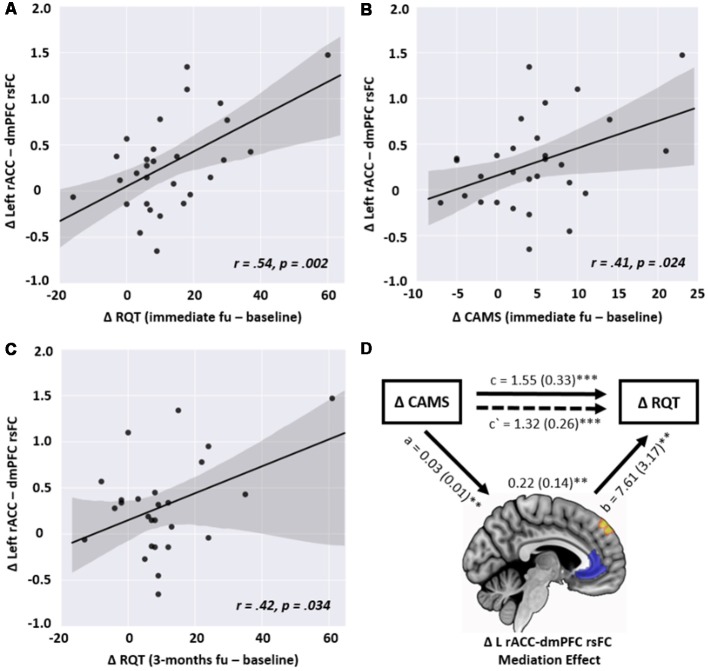
Scatterplots of the relationship between the changes in left rACC—dmPFC resting-state functional connectivity (rsFC) and the changes in the following psychological measures in the meditation group: **(A)** changes in the Resilience Quotient Test (RQT) between baseline and immediate follow-up (*r* = 0.54, *p* = 0.002), **(B)** changes in the Cognitive and Affective Mindfulness scale (CAMS) between baseline and immediate follow-up (*r* = 0.41, *p* = 0.024), **(C)** changes in RQT between baseline and 3-month follow-up (*r* = 0.42, *p* = 0.034), **(D)** the positive association between the changes in CAMS (*X*) and RQT (*Y*), mediated by the changes in left rACC-dmPFC rsFC (*M*), showing that changes in rsFC partially contribute to the prediction of resilience enhancement. *a*, *b*, and *c* are path coefficients. **p < 0.01, ***p < 0.001.

In the correlation analysis, the changes in the left rACC-left dmPFC connectivity in the MED group were significantly correlated with the changes in RQT and CAMS (*r* = 0.54, *p* = 0.002 and *r* = 0.41, *p* = 0.024, respectively) in terms of the short-term effect. However, this correlation was not observed in the control group. No other type of rsFC from the GLM analysis was significantly associated with psychological measures. Concerning the long-term effect, the change in the rsFC was still significantly correlated with improved RQT (*r* = 0.42, *p* = 0.034) but not for CAMS (*r* = 0.309, *p* = 0.124; Figure 4).

As shown in Figure 4, the mediation analysis revealed a significant partially mediating effect on the rACC-dmPFC rsFC through the relationship between CAMS and RQT. In other words, the coefficient between X and Y was decreased when the change in the rACC-dmPFC rsFC was included (*c* = 1.55, *p* < 0.001; *c*′ = 1.32, *p* < 0.001). Significant relationships were also observed for the path coefficients between *X* and *M* (*a* = 0.03, *p* = 0.006) and between *M* and *Y* (*b* = 7.61, *p* = 0.003).

### Effect of Religion

To investigate the effect of the participants’ religious beliefs on our results, ANOVAs and independent sample *t*-tests were conducted for the MED group. There were no significant differences in the baseline of rACC-dmPFC rsFC (*F*_(1,25)_ = 0.81, *p* = 0.498), CAMS (*F*_(1,25)_ = 1.67, *p* = 0.199), or RQT (*F*_(1,25)_ = 2.59, *p* = 0.075) depending on the participants’ religions. In addition, there were no significant differences detected between participants with religion and those without religion in terms of baseline of rACC-dmPFC rsFC (*t* = −0.41, *p* = 0.68), CAMS (*t* = 0.12, *p* = 0.90), or RQT (*t* = −1.25, *p* = 0.808). In terms of the changes at the immediate follow-up, there were no significant differences in the changes in rACC-dmPFC rsFC (*F*_(1,25)_ = 1.203, *p* = 0.328), CAMS (*F*_(1,25)_ = 0.925, *p* = 0.443), or RQT (*F*_(1,25)_ = 0.121, *p* = 0.947) depending on the participants’ religion. In addition, there were no significant differences detected between participants with religion and those without religion in terms of changes in rACC-dmPFC rsFC (*t* = −0.93, *p* = 0.36), CAMS (*t* = 0.57, *p* = 0.57), or RQT (*t* = 1.70, *p* = 0.10).

## Discussion

To our knowledge, this is the first highly controlled residential study to investigate the neural changes *via* short-term intensive meditation, which is associated with the enhancement and maintenance of resilience. Our results revealed that the DMN was significantly enhanced in the MED group after the intervention compared with that in the control group. In particular, the changes in the functional connectivity of the left rACC with the dmPFC were correlated with the change in resilience at both the immediate and 3-month follow-ups in the MED group. In addition, mediation analysis revealed that the change in the left rACC-dmPFC functional connectivity was a partial mediating factor in the association between the enhancement in mindfulness and resilience immediately after the intervention. Our results suggest that the increase in the rACC-dmPFC rsFC was not only related to the immediately enhanced level of resilience but also predicted the long-term maintenance of the improvement.

Consistent with previous studies, compared with the control group, the MED group showed significantly increased rsFC between the left rACC and dmPFC, precuneus and angular gyrus (Brewer et al., 2011; Jang et al., 2011; Hasenkamp et al., 2012; Prakash et al., 2012; Taylor et al., 2012; Garrison et al., 2013). Since the precuneus, angular gyrus, and dmPFC are the functional hubs of DMN, we suggest that there was an increase of DMN in the MED group after meditation (Andrews-Hanna et al., 2014). Among the regions, the rACC-dmPFC rsFC was specifically associated with the resilience improvement *via* meditation. Such changes in rACC and dmPFC activation have been observed consistently in previous meditation studies (Farb et al., 2007; Hölzel et al., 2007). Regarding the roles of the rACC and dmPFC in the monitoring of an individual’s affective state and in mentalizing, rACC-dmPFC connectivity may reflect individuals’ abilities to understand the mental state of self (Milham and Banich, 2005; Lombardo et al., 2010). As a central feature of Buddhist meditation, mentalization enhances self-awareness with mindfulness practices (Koriat, 2000). Moreover, it is considered a key component in strengthening individuals’ resilience and shares some of the underlying neural mechanisms (Stein, 2006). Taken together, our results suggested that changes in rsFC in the regions related to mentalizing and resilience caused by mindfulness practice facilitated the short and long-term improvements of resilience in the MED group.

At the 3-month follow-up, the improvement in the resilience scores was significantly maintained in the MED group compared with that in the control group. Resilience is known to be relevant to the onset, recurrence, and symptom severity of various psychiatric disorders, including depression and anxiety disorders (Ludwig and Kabat-Zinn, 2008; Segal et al., 2010; Goyal et al., 2014). For this reason, many adjunctive therapies have been constantly developed to improve resilience and have proven to be effective (Fava and Tomba, 2009; Padesky and Mooney, 2012). However, only the meditation intervention, along with a few other interventions, has been shown to be effective within the short term and is self-trainable without an instructor after appropriate professional training. Furthermore, our results suggest that improved resilience *via* short-term meditation is maintained for up to 3 months. This evidence supports the hypothesis that short-term meditation might be a useful adjunctive intervention for individuals at high risk of psychiatric disorders. Moreover, the changes in the rACC-dmPFC rsFC may predict the maintenance effects of adjunctive therapy.

Interestingly, both groups demonstrated significant enhancements in psychological variables at the immediate follow-up, but the changes in rACC-dmPFC rsFC show different patterns between the groups. These results suggest that each group adopted different underlying mechanisms beyond the increased resilience and mindfulness scores. In the MED group, intensive meditation training seems to facilitate the resilience-related network, which eventually led to the psychological enhancement and its maintenance. In contrast, the changes in psychological measures in the control group were not able to show either significant correlation with rsFC or the maintenance of the scores. We suspect that their temporarily increased scores are due to the escape from everyday stress and relaxation rather than to the improvement in resilience.

In the present study, the control group was also exposed to interventions, such as escape from everyday stress, changes in environment and diet, and encountering new people. Such a design was based on an effort to minimize non-meditation factors, but our results suggest that the intervention also causes neural and behavioral changes. These changes may imply a “vacation effect,” as reported in previous studies (de Bloom et al., 2009; Epel et al., 2016). The vacation effect refers to the temporary improvements in health and psychological well-being after taking a vacation, which soon fades after work resumption. The absence of the maintenance effect of psychological measures in the control group also supports this definition. Unfortunately, we are unable to identify the exact factor that caused the changes in the rsFC of the control group after intervention because of the lack of any correlation with psychological measures. Moreover, randomized controlled studies to clarify the effect of vacation on neural levels, especially in resting-state connectivity, are very rare. In further studies, it will be necessary to explore the rsFC changes in the control group in more depth with various psychological measures, larger sample size and more follow-up time points.

We suspect a few possible few causes for the rsFC changes detected in the control group after the intervention. First, detachment from stress and everyday life may cause participants to feel relieved, which eventually led to changes in self-reported questionnaires and rsFC. Previous studies reported reduced rACC-temporal gyrus rsFC in veterans whether the individuals had PTSD or not (Kennis et al., 2015). The study suggested that experiencing stressful situations may alter the neural network. Unfortunately, we are unable to support the further explanation of the increased the rACC-temporal gyrus in the control because of the lack of correlation with psychological variables, but we suspect that the changes in the neural network were related to stress relief. Second, we cautiously assume that the diary writing in the control group might have enhanced language-related connectivity after the intervention. Some studies also demonstrated the effects of diary training at the cognitive and behavioral levels (Ho et al., 2011; Travers, 2011). The fronto-temporal rsFC has been known for its role in the language network; in particular, decoupling between ACC-temporal gyrus connectivity is associated with auditory hallucinations in individuals with schizophrenia (Stamatakis et al., 2005). However, it might be premature to suggest that writing in diaries account for the rsFC changes in the control group since previous studies used weeks of diary training. In the current study, further explanations of the rsFC changes in the control group are limited, but we suggest that these changes are due to the environmental modifications, including residence, diet, and other lifestyles.

The present study exhibits several strengths in exploring the effect of meditation exclusively. First, we recruited participants without prior experience in meditation or Templestay; therefore, all participants underwent the same period of intervention. Second, this study was designed as a longitudinal study and a randomized controlled trial to reduce possible pre-existing individual differences in the characteristics and brains of the participants. Third, we included an active control group to reduce the expectation effect on the participants. Finally, all of the participants, regardless of their group, were exposed to the same environment and diet in a retreat format to minimize any non-meditation-related factors.

When compared with previous studies, although the present study exhibits the above strengths, it also presents some potential limitations that should be noted. First, the individual level of mindfulness and resilience were assessed using self-reported questionnaires. Therefore, the scores may include some subjective bias. However, these scores have been used in many studies, and no objective tool for measuring these factors has been invented to date. Second, some participants’ religion was Buddhism, and there is a possibility that their religion may have an effect on the result, even though they reported a lack of prior experience in meditation or Templestay. However, our statistical analyses support that the effect of religion was not statistically significant at either the baseline or in the changes in self-reported questionnaires or in the rsFC. Third, even though we assessed self-reported questionnaires at three time points (baseline, an immediate follow-up after the intervention, and a 3-month follow-up), we acquired brain imaging data only at baseline and at the immediate follow-up due to the participants’ schedules and financial issues. For these reasons, it is unknown whether the change in neural connectivity was also maintained after 3 months. Further studies are needed to confirm the maintenance of neural changes and their relationships with other beneficial effects of meditation. In addition, more refined parcellation of ACC is recommended for the future studies, especially those with mental health conditions. Many recent studies suggest a distinct role of subgenual region and pregenual region of perigenual cingulate cortex, and different degrees of deficits in individuals with various psychiatric disorders (Davey et al., 2012; Zeng et al., 2014a). Adaptation of more elaborated ROI may clarify the effect of meditation in the neural level.

In conclusion, our highly controlled residential trial involving 4 days of meditation practice generated findings worthy of further study. Our study indicates that changes in the rACC-dmPFC rsFC may mediate improvement in and maintenance of individuals’ resilience *via* short-term meditation. Given that resilience is a crucial factor in mental health conditions, our results may contribute to intervention and prevention programs for individuals at risk of psychiatric disorders. Moreover, the rACC-dmPFC rsFC may be a potential neural target for future adjunctive therapies.

## Data Availability

The datasets generated for this study are available on request to the corresponding author.

## Author Contributions

SK: acquisition of data, analysis, manuscript interpretation, making figures, revision and final approval of the article, agreement to be accountable for all aspects of the work. TL, WJ and J-WH: interpretation, critical comments on manuscript, revision and final approval of the article, agreement to be accountable for all aspects of the work. DB, KC and WH: acquisition of data, revision and final approval of the article, agreement to be accountable for all aspects of the work. K-OL, S-YK, HP and JK: acquiring fund, conception and design of the work, interpretation, critical comments on manuscript, revision and final approval of the article, agreement to be accountable for all aspects of the work.

## Conflict of Interest Statement

The authors declare that the research was conducted in the absence of any commercial or financial relationships that could be construed as a potential conflict of interest.

## Footnotes

^1^https://fsl.fmrib.ox.ac.uk/fsl/

^2^http://github.com/canlab/MediationToolbox
