# Supplementary material for: The Immediate and Sustained Positive Effects of Meditation on Resilience Are Mediated by Changes in the Resting Brain
Source: Front Hum Neurosci. 2019 Mar 26;13:101. doi: 10.3389/fnhum.2019.00101 (PMC6448020; doi:10.3389/fnhum.2019.00101)
Supplement: Supplementary file 1 [file Table_1.DOCX]

**Supplementary Appendix**

Supplement to: **“The immediate and sustained positive effects of meditation on resilience are mediated by changes in the resting brain”**

**Table of Contents**

Supplementary Methods 2

Supplementary References 5

Supplementary Figures 6

Figure S1. Flowchart of the intervention study, including enrollment, randomization and retention 6

Figure S2. The effect of each intervention on functional connectivity 7

**Supplementary Methods**

***Intervention (Templestay program)***

All sessions in both the MED and control groups were conducted during a single 4-day, 3-night residential retreat at the temple of Daewonsa (eng.templestay.com) in South Korea. The program for the MED group focused on training for the lifestyle of the Buddhist monk, including mindfulness meditation, mindful eating, walking, and thinking. A total of 19 hours of the meditation practice indicated above were involved in the meditation intervention. In contrast, the control group was instructed to rest at the temple, including walking around the temple and mountain and interacting with other participants in the session. In addition, they were required to write a diary of the activities they performed and to reflect on the day. The control group was not exposed to any meditation-related activities in the temple, and a research assistant accompanied the participants to monitor their activities.

The two groups were provided with the same residence areas and meals but were not present at the temple at the same time. Data were collected from July 2014 to July 2015, from a total of 12 sessions (7 sessions for the MED group and 5 sessions for the control group). More detailed information on the program and the changes induced by the intervention is provided in our previous report^1^.

Supplementary References

1. Hwang, W. J. *et al.* The effects of four days of intensive mindfulness meditation training (Templestay program) on resilience to stress: A randomized controlled trial. *Psychol. Health Med.* **23**, 497-504 (2018).

Supplementary Figures


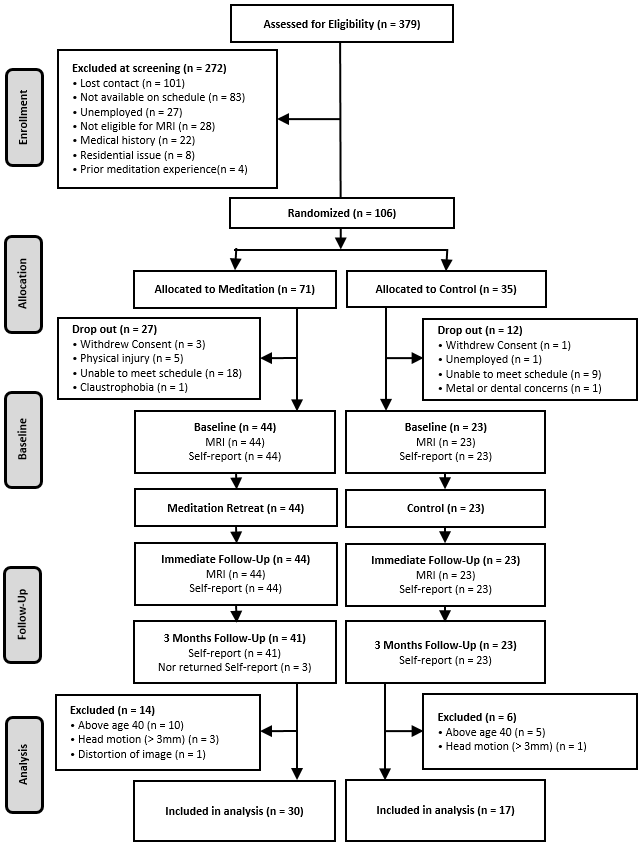


**Figure S1. Flowchart of the intervention study, including enrollment, randomization and retention.** The numbers of participants dropped out or excluded at each stage were listed with reasons. Of the 379 volunteers, 106 were eligible (28%). After random assignment to either group using a computerized algorithm, 39 were dropped out (38%) due to above reasons. In this study, 30 participants in the meditation group and 17 participants in the control group were included with above exclusion criteria.


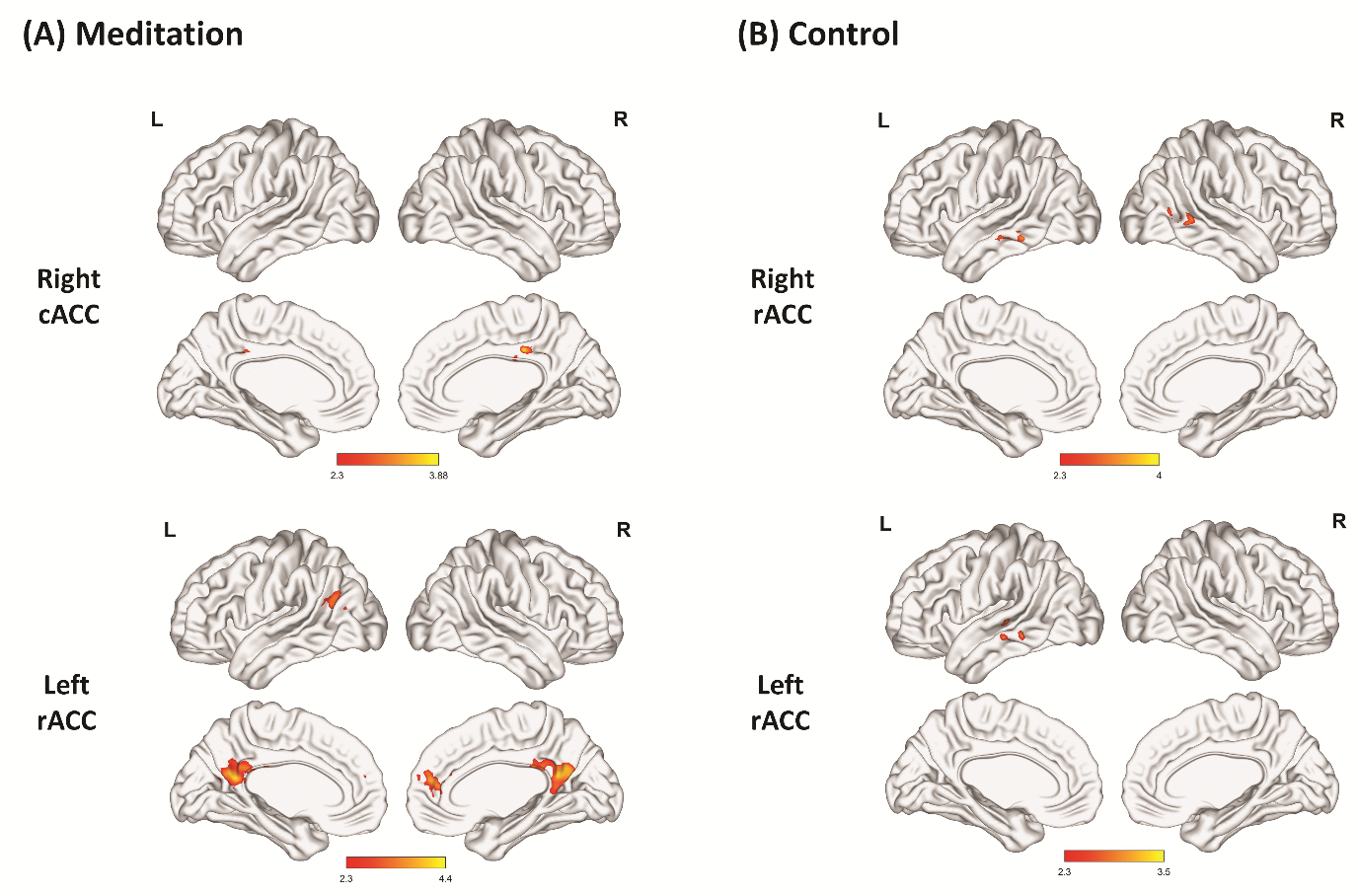


**Figure S2. The effect of each intervention on functional connectivity.** (A) In the meditation group, significant increases in the regions involved in the DMN, (B) In the control group, significant increases in the bilateral rACC rsFC with the temporal gyrus.
